# A Nursing-Focused Quasi-Experimental Study on Compressive Cryotherapy for Postoperative Recovery in Knee Arthroscopy Patients

**DOI:** 10.3390/jcm15020586

**Published:** 2026-01-11

**Authors:** Ibrahim Alasqah, Mona Metwally El-Sayed, Helalia Shalabi Mohamed Shalab, Mahmoud Abdelwahab Khedr

**Affiliations:** 1 Department of Community, Psychiatric, and Mental Health Nursing, College of Nursing, Qassim University, Buraydah 52571, Saudi Arabia; i.alasqah@qu.edu.sa; 2 Department of Community Health Nursing, Faculty of Nursing, Cairo University, Cairo 12613, Egypt; hs.shalabi@paaet.edu.kw; 3 College of Nursing, Public Authority for Applied Education and Training (PAAET), Kuwait City 70466, Kuwait; 4 Department of Psychiatric and Mental Health Nursing, Faculty of Nursing, Alexandria University, Alexandria 21527, Egypt; mahmoud.khedr@alexu.edu.eg

**Keywords:** arthroscopy, knee joint, patient care, postoperative period, cryotherapy

## Abstract

**Background:** Compressive cryotherapy, which combines cold therapy with compression, has gained attention to relieve pain and swelling after the Knee arthroscopy. However, there is still limited evidence specifically related to its use after knee arthroscopy. **Objective:** This study investigated the efficacy of compressive cryotherapy in decreasing postoperative pain and swelling in patients following knee arthroscopy. **Methods:** A quasi-experimental study was conducted at the Kasr Al-Ainy Hospital. Sixty patients scheduled for knee arthroscopy were divided into two groups. The intervention group (*n* = 30) received compressive cryotherapy using a cold-pack knee wrap set at 2 to 5 °C for 15 to 20 min, three times daily. The control group (*n* = 30) received standard postoperative care. Pain was assessed with the Numerical Rating Scale. Swelling was measured by assessing knee circumference at the mid-patella. Assessments occurred immediately after surgery (baseline), and on the first and second postoperative days. Non-parametric tests used in the analysis included the Chi-square test, the Mann–Whitney U test, the Friedman test, and the Wilcoxon signed-rank test with Bonferroni–Holm correction. **Results:** Patients in the compressive cryotherapy group experienced a greater reduction in pain than those in the control group. By the first postoperative day, none of the patients in the intervention group reported severe pain (*p* < 0.001). Knee circumference decreased significantly in the intervention group, from a median of 51.05 cm [IQR: 49.1–53.2] at baseline to 40.90 cm [39.8–42.1] by the second day. In comparison, the control group showed a smaller reduction, from 52.70 cm [50.8–54.5] to 48.55 cm [46.8–50.9]. Between-group differences in swelling were significant at the first postoperative assessment (U = 105.0, *p* < 0.001) and on day 2 (U = 62.5, *p* < 0.001). Overall, differences in both pain intensity and knee swelling between groups were statistically significant across all time points (*p* < 0.001). **Conclusions:** Compressive cryotherapy is an effective non-pharmacological intervention for reducing pain and swelling in the early postoperative period following knee arthroscopy. These results suggest that it could be a valuable addition to routine postoperative care, helping patients recover more comfortably and quickly.

## 1. Introduction

Knee joint injuries are closely linked to fatigue and ongoing microdamage of ligament tissues caused by repetitive loading over time [1,2]. This cyclic mechanical stress can lead to microscopic degeneration, decrease ligament stiffness, and cause structural failure at stresses lower than the tissue’s single-load capacity. During daily and sports activities, knee ligaments face complex combinations of tensile, shear, and torsional forces, and prolonged exposure to these loads can gradually weaken their structural integrity, increasing the risk of acute rupture or chronic joint instability [1,3].

Arthroscopic knee (AK) surgery is one of the most frequently performed orthopedic procedures worldwide, with an estimated 2 million knee arthroscopies carried out annually, particularly in high-income countries. Despite recent guideline-driven reductions in arthroscopy for degenerative osteoarthritis, utilization remains substantial, especially among middle-aged adults, who constitute a large proportion of the working-age population undergoing procedures for meniscal and other intra-articular knee disorders [4].

Despite its minimally invasive nature and widespread use, recovery following arthroscopic knee surgery can remain challenging for many patients. During the early postoperative period, patients commonly experience postoperative pain, swelling, and inflammatory responses. These symptoms may affect not only the knee joint but also the surrounding soft tissues, thereby complicating rehabilitation. If inadequately managed, such postoperative complications can hinder recovery and delay patients’ return to normal daily activities [5].

Although AK surgery is less invasive than open procedures, it can still lead to notable postoperative challenges. Many patients experience significant knee pain and swelling in the immediate postoperative phase. These symptoms result from several factors inherent to the surgical procedure, including manipulation of sensitive anatomical structures such as the fat pad, joint capsule, and anterior synovium [6]. Additionally, activating nociceptors and free nerve terminals within the synovial tissue contributes to the pain experience. The body’s inflammatory response to surgical tissue trauma further exacerbates local swelling [7]. The management of these postoperative symptoms is crucial, as uncontrolled pain and swelling can have far-reaching consequences. Patients may face extended hospital stays, delayed recovery timelines, and increased utilization of medical resources if these issues are not adequately addressed. This underscores the importance of effective postoperative care strategies [8].

Cryotherapy, commonly referred to as “cold therapy,” has gained widespread popularity as a non-pharmacological intervention in postoperative care, particularly following knee arthroscopy [9]. This therapeutic approach aims to reduce local tissue metabolism by lowering tissue temperature. The primary goals of cryotherapy after knee arthroscopy include pain relief, reduction in edema, minimization of bleeding, and improvement in joint range of motion [10]. Modern cryotherapy applications can be categorized into two main types: dry and wet. Dry cold applications typically involve ice packs or cold gel packs, while wet, cold applications encompass methods such as ice or cold water-soaked compresses, ice massage, and localized or general cold water baths [11].

Compressive cryotherapy represents an advanced form of this treatment modality. This approach combines the benefits of cold therapy with compression and can be administered through various means, including ice packs, cold-compression braces, or sophisticated computer-assisted cryotherapy devices. The physiological mechanisms underlying compressive cryotherapy are multifaceted. It works by lowering the intra-articular temperature and slowing nerve conduction velocity, thereby limiting pain perception. Additionally, it induces immediate vasoconstriction, reduces vascular spasm, and decreases blood flow to the affected area, thereby contributing to the reduction in tissue edema [12].

A key advantage of combining compression with cold therapy is enhanced contact between the cooling agent and the skin. This improved contact increases thermal conduction, thereby amplifying the effect of cold application on tissue temperature [13]. Moreover, the compression element further reduces blood flow to the treated area. This synergistic effect enhances the overall therapeutic benefit and may accelerate recovery by more effectively managing postoperative pain and swelling [9].

Integrating compressive cryotherapy into postoperative care protocols for knee arthroscopy patients represents a promising approach to enhancing recovery outcomes. By leveraging the combined effects of cold therapy and compression, this technique offers a comprehensive strategy for managing patients’ common challenges in the immediate postoperative period [14]. As research in this area continues to evolve, compressive cryotherapy may become an increasingly important component of evidence-based postoperative care in orthopedic settings.

Recent studies have highlighted the potential benefits of compressive cryotherapy in managing postoperative pain and inflammation, particularly following arthroscopic knee surgery [11,14]. This advanced form of cryotherapy, which combines cold therapy with compression, has shown promise in enhancing the recovery process compared to traditional cryotherapy methods alone [15]. However, the evidence supporting the efficacy of compressive cryotherapy specifically for reducing pain and swelling following knee arthroscopy remains limited, indicating a significant gap in our understanding of its potential benefits in this surgical context.

Several studies have suggested that compressive cryotherapy may offer superior outcomes in the early stages of rehabilitation after various knee surgeries [10,16]. These findings have sparked interest in the orthopedic community, as effective management of postoperative pain and swelling is crucial for optimizing patient recovery and facilitating early mobilization. Despite these promising indications, the application of compressive cryotherapy in the post-arthroscopic knee surgery setting has not been thoroughly investigated, leaving clinicians with uncertainty regarding its effectiveness in this patient population.

More recently, a 2023 meta-analysis of seven randomized trials involving 519 patients compared continuous cryotherapy with traditional cryotherapy after total knee arthroplasty and found no significant differences between the two approaches in postoperative pain, analgesic use, range of motion, knee swelling, blood loss, hemoglobin change, or transfusion rates. There were also no clinically important differences in length of hospital stay or adverse events, whereas continuous cryotherapy demanded greater financial and logistical resources than standard cold therapy [17].

The limitations in previous research underscore the need for a focused investigation into the effects of compressive cryotherapy on patients undergoing knee arthroscopy. Specifically, there is a pressing need to evaluate its impact on two vital postoperative challenges: pain intensity and swelling. These factors are critical determinants of patient comfort, rehabilitation progress, and surgical outcomes. By addressing this knowledge gap, healthcare providers can make more informed decisions about incorporating compressive cryotherapy into postoperative care protocols for arthroscopic knee surgery patients.

Accordingly, the present study aimed to comprehensively evaluate the efficacy of compressive cryotherapy on pain intensity and swelling in patients following knee arthroscopy. By focusing on these specific outcomes, the research seeks to contribute valuable insights to the growing evidence surrounding postoperative care in orthopedic surgery. This study’s findings inform clinical practice, potentially leading to improved pain management strategies and enhanced recovery protocols for patients undergoing arthroscopic knee procedures.

## 2. Research Hypotheses

**H1.**  *Patients who have undergone knee arthroscopy and receive compressive cryotherapy would experience a more significant reduction in postoperative pain intensity compared to those who do not receive this intervention.*

**H2.**  *Patients who have undergone knee arthroscopy and receive compressive cryotherapy would experience a more significant reduction in postoperative knee swelling than those who do not receive this intervention.*

## 3. Methods

### 3.1. Research Design

A quasi-experimental research design was employed under the Transparent Reporting of Evaluations with Nonrandomized Designs (TREND) guidelines [18]. Following the acquisition of administrative approval, data collection commenced over ten months, from the end of August 2024 to the end of December 2024.

### 3.2. Setting

The study was conducted at the Kasr Al-Ainy Department of Orthopedic and Trauma Surgery Hospital, Cairo University. The hospital is a tertiary care center specializing in orthopedic and trauma services, equipped with modern facilities and a dedicated orthopedic department. According to the hospital’s statistical census, it has approximately 521 beds and serves a diverse patient population with a wide range of musculoskeletal conditions.

### 3.3. Target Participants and Sample Size Estimation

This study included patients scheduled for elective knee arthroscopy. The required sample size was determined a priori using G*Power software (version 3.1.9.7) based on an independent-samples *t*-test [19,20]. The calculation assumed a medium effect size (Cohen’s d = 0.5) [21] for the primary outcome of postoperative pain reduction, informed by previous meta-analyses evaluating the effects of compressive cryotherapy following knee surgery [22,23]. Statistical parameters were set at a power (1 − β) of 0.95 and a two-tailed alpha (α) level of 0.05. The analysis indicated a minimum sample size of 54 participants (27 per group). To account for an anticipated attrition rate of approximately 20%, the total sample size was increased to 60 participants, with 30 allocated to the intervention group and 30 to the control group.

### 3.4. Eligibility Criteria

Patients aged 18–65 years who underwent elective knee arthroscopy for conditions such as meniscal tears or ligament repair were eligible for inclusion. Participants were required to be within 24 h postoperatively at the time of enrollment, capable of understanding and following verbal and written instructions related to compressive cryotherapy, and willing to provide written informed consent. Patients were excluded if they had significant comorbidities that could adversely affect postoperative recovery (e.g., uncontrolled diabetes mellitus or cardiovascular disease), required intensive postoperative care, or had known contraindications or hypersensitivity to cold therapy or materials used in compressive cryotherapy, including latex. Additional exclusion criteria included postoperative complications such as deep vein thrombosis or pulmonary embolism, inflammatory or neoplastic conditions associated with persistent pain, and neurological disorders affecting sensation or mobility of the lower extremities.

### 3.5. Sampling and Recruitment

**Figure 1:**
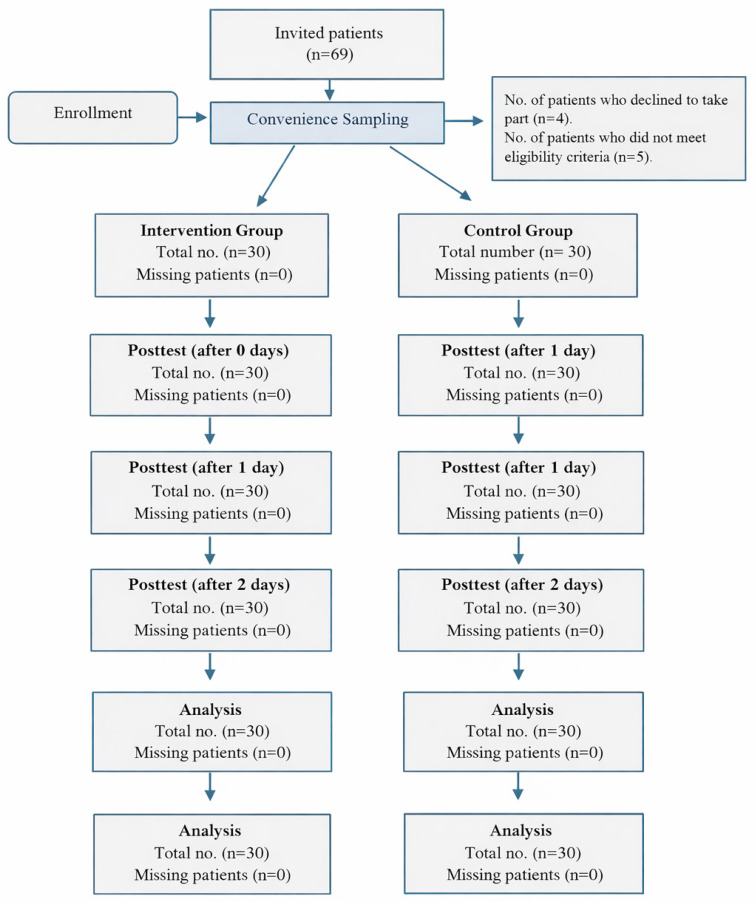
Participants’ Recruitment.

A non-probability convenience sampling method was used to recruit patients scheduled for knee arthroscopy into the intervention and control groups. Initially, 69 patients were assessed for eligibility. Of these, 4 declined to participate, and 5 were excluded based on the predefined eligibility criteria. Consequently, the final study sample comprised 60 participants, with 30 allocated to the intervention group and 30 to the control group. Participants in the control group were recruited first, underwent baseline assessment, and completed post-intervention outcome measures. Subsequently, participants were recruited into the intervention group (Figure 1).

### 3.6. Control Group and Standard Care

Participants in the control group received standard postoperative care following knee arthroscopy, referred to as treatment as usual (TAU), which was tailored to each patient’s clinical condition. Standard care included routine pharmacological management, such as antibiotics and analgesics, including cefazolin (1 g intravenously administered preoperatively) and ibuprofen (400 mg every 8 h as needed). Follow-up assessments were performed immediately after surgery and on the first and second postoperative days, without the application of any additional interventions specifically aimed at reducing pain or postoperative symptoms.

### 3.7. Participant Matching and Control for Confounding

A quasi-experimental design was used due to practical clinical constraints that made full randomization unfeasible. However, specific measures were taken to reduce the risk of selection bias. Participants were enrolled sequentially, with the control group recruited first, followed immediately by the intervention group. This method was intended to minimize variations in care delivery over time. All participants were selected using the same strict eligibility criteria to ensure consistency. Baseline demographic and clinical characteristics were compared between the two groups, and no statistically significant differences were found (*p* > 0.05; see Table 1). To further account for potential residual confounding, sensitivity analyses were conducted using Analysis of Covariance (ANCOVA), adjusting for baseline values of the outcome measures.

### 3.8. Study Measurements

Three outcome measures were used to collect data in accordance with the study objectives.

### 3.9. Demographic and Clinical Data Sheet

A structured questionnaire was used to collect demographic and clinical information from participants, including age, gender, marital status, educational level, occupation, region of residence, and smoking history. Additional data included past medical history, previous knee surgeries, and the indication for the current admission.

### 3.10. Numerical Rating Scale-11 (NRS-11)

The Numerical Rating Scale–11 (NRS-11) was used to assess self-reported pain intensity. The scale consists of an 11-point range from 0 (no pain) to 10 (worst pain imaginable), with participants selecting the number that best reflects their current pain level. Pain intensity was categorized as no pain (0), mild pain (1–3), moderate pain (4–6), and severe pain (7–10). The Arabic version of the NRS-11 has demonstrated good test–retest reliability, with a reported coefficient of 0.82 [23].

### 3.11. Knee Circumference Measurements (cm)

Knee circumference was measured at the mid-patellar level with the knee in full extension while the patient was seated. A single trained researcher, blinded to group allocation, performed all measurements using a flexible tape measure (1.5 cm wide, 1 m long, marked in 1 mm increments). To ensure intra-rater reliability, duplicate measurements were taken at each time point, and the mean value was recorded. The intra-class correlation coefficient (ICC) for repeated measurements was 0.93, indicating excellent reliability [24,25].

## 4. Procedure

### 4.1. Ethical Approval

The study was conducted in accordance with the Declaration of Helsinki (DoH, October 2008) and approved by the Research Ethics Committee (REC) of the Faculty of Nursing, Cairo University (IRB000589). Written permission was obtained from the hospital administration prior to data collection. All participants provided written informed consent after receiving a clear explanation of the study objectives and procedures. Participation was voluntary, with the right to withdraw at any time, and participant confidentiality was strictly maintained.

### 4.2. Pilot Study

A pilot study was conducted at the beginning of the research to assess the feasibility and applicability of the data collection tools. The pilot included 10 patients, who were not included in the final study sample.

### 4.3. Data Collection

#### 4.3.1. Preoperative Phase

Baseline demographic and clinical data were collected from both the control and intervention groups during the preoperative period. Each participant completed a structured interview lasting approximately 20–25 min. Body mass index (BMI) was calculated by a trained researcher using measurements obtained with the clinic scale, expressed as weight in kilograms divided by height in meters squared (kg/m^2^).

#### 4.3.2. Postoperative Phase

In the intervention group, compressive cryotherapy was initiated on the day of surgery and continued for the first two postoperative days. The intervention was delivered using a standardized cold-pack knee wrap applied over a single layer of dry surgical dressing. Cold packs were maintained at a temperature of 2–5 °C, verified before each session using a calibrated digital infrared thermometer. The knee wrap was secured to provide consistent, moderate circumferential compression, assessed as snug but non-restrictive, with preserved distal capillary refill (<2 s). Each treatment session lasted 15–20 min and was administered three times daily at two-hour intervals. The protocol was designed to ensure effective delivery of therapeutic cooling and compression while minimizing the risk of cold-related injury or vascular compromise.

### 4.4. Evaluation and Follow-Up

Pain intensity and knee circumference were assessed according to a predefined timeline. Baseline measurements (T0) were obtained immediately after surgery and prior to the application of any study intervention to establish a postoperative baseline. In the intervention group, an additional pain assessment was performed 15 min after the first compressive cryotherapy session on the day of surgery to evaluate the immediate analgesic effect. Follow-up assessments were conducted on the first (T1) and second (T2) postoperative days. These measurements were obtained in the morning, before the scheduled cryotherapy session for the intervention group, to assess sustained treatment effects and postoperative progression.

### 4.5. Data Statistical Analysis

Data analysis was performed using IBM SPSS Statistics for Windows, Version 26.0 (IBM Corp., Armonk, NY, USA). The normality of all continuous variables was assessed using the Kolmogorov–Smirnov and Shapiro–Wilk tests. Since the primary outcome measures, including ordinally categorized pain intensity and knee circumference, did not meet parametric assumptions (*p* < 0.05), a comprehensive non-parametric analytical strategy was adopted. Intergroup comparisons of demographic characteristics and pain intensity categories at each postoperative time point were conducted using the Chi-square (χ^2^) test, with the Monte Carlo simulation method applied for contingency tables containing expected cell frequencies below five. Between-group comparisons of knee circumference at each assessment point (T0, T0 + 15 min, T2) were performed using the Mann–Whitney U test, while within-group changes in circumference over time were evaluated with the Wilcoxon signed-rank test, applying the Bonferroni–Holm correction for multiple pairwise comparisons. For ordinal repeated measures, intragroup changes in pain intensity across postoperative assessments were analyzed using the Friedman test; statistically significant results were further examined with post hoc pairwise Wilcoxon signed-rank tests, adjusted via the Bonferroni–Holm procedure. All inferential tests were two-tailed, with statistical significance set at *p* < 0.05.

## 5. Results

Most participants in both groups were aged 40–50 years, with 36.7% in the study group and 33.3% in the control group, with no significant difference (χ^2^ = 0.154, *p* = 0.878). Regarding gender, the study group was 46.7% male and 53.3% female, while the control group was 33.3% male and 66.7% female; the difference was not significant (χ^2^ = 2.443, *p* = 0.192). Educational levels varied, with more illiterate individuals in the study group (56.7%) than in the control (40.0%), but this was insignificant (χ^2^ = 3.973, *p* = 0.290). Both groups had similar marital status (56.7% married), and differences in other statuses were not significant (χ^2^ = 2.9, *p* = 0.435). Unemployment was 46.7% in the study group and 33.3% in controls, not significant (χ^2^ = 1.314, *p* = 0.518). Residence was evenly split (30% rural, 70% urban), with no difference (χ^2^ = 0.000, *p* = 1.000). Most reported insufficient income (60% in the study, 56.7% in controls), with no significant difference (χ^2^ = 3.354, *p* = 0.067). Demographically, both groups were comparable. Both had 36.7% with comorbidities (χ^2^ = 0.000, *p* = 1.000). Diagnosis via knee arthroscopy was similar, with 26.7% and 30.0% for diagnostic procedures, and most had therapeutic procedures, no significant difference (χ^2^ = 0.000, *p* = 1.000). Prior knee surgeries were reported by 30.0% in the study group and 26.7% in controls, with no significant difference (χ^2^ = 0.082, *p* = 0.774). Family history of orthopedic disease was 40.0% in both groups (χ^2^ = 0.000, *p* = 1.000). BMI classifications showed similar distributions: average weight (20.0% vs. 13.3%), overweight (26.7% vs. 30.0%), and obese (53.3% vs. 56.7%), with no significant differences (χ^2^ = 0.622, *p* = 0.733). Overall, clinical characteristics were comparable across parameters, as shown in Table 1.

Immediately after surgery (T0, baseline), all participants in both groups reported severe pain. After the first application of compressive cryotherapy (T0 + 15 min), only 26.6% of the intervention group still reported severe pain, compared to 80.0% in the control group (*p* < 0.001). On the first postoperative day (T1), no patient in the intervention group reported severe pain, while 70.0% of the control group did (*p* < 0.001). By the second postoperative day (T2), the intervention group reported predominantly mild pain (56.7%), whereas 46.7% of the control group still experienced severe pain (*p* < 0.001). Within-group analysis using the Friedman test showed significant reductions in pain severity over time in both groups (*p* < 0.001), as presented in Table 2.

Between-group comparisons of knee circumference at each postoperative time point were analyzed using the Mann–Whitney U test. At baseline (T0), no significant difference was observed between the intervention and control groups (U = 382.5, *p* = 0.390). However, following the first compressive cryotherapy session (T0 + 15 min), knee circumference was significantly lower in the intervention group (median [IQR]: 42.20 cm [40.5–44.1]) compared to the control group (50.10 cm [48.3–52.0]; U = 105.0, *p* < 0.001). This significant reduction was maintained on the second postoperative day (T2), with the intervention group measuring 40.90 cm [39.8–42.1] versus 48.55 cm [46.8–50.9] in the control group (U = 62.5, *p* < 0.001). Within-group changes over time were assessed using the Friedman test, which indicated significant reductions in knee circumference across time points in both the intervention group (χ^2^(2) = 58.32, *p* < 0.001) and the control group (χ^2^(2) = 28.15, *p* < 0.001). Post hoc Wilcoxon signed-rank tests with Bonferroni–Holm correction confirmed significant pairwise reductions at all time comparisons (*p* < 0.005 for all), demonstrating a more pronounced and sustained decrease in swelling in the compressive cryotherapy group, as shown in Table 3.

## 6. Discussion

Although knee arthroscopy is regarded as a slightly invasive procedure, patients often face short-term postoperative issues such as pain, swelling, and limited function. Managing these symptoms effectively in the early recovery phase is crucial for improving healing and encouraging early restoration of movement [3,4,25]. Compressive cryotherapy has become popular as a non-drug treatment that merges cold therapy with compression to reduce postoperative pain and swelling [11,26]. Despite its increasing clinical application, evidence about its effectiveness after knee arthroscopy remains limited. Therefore, this study aimed to fill that gap by examining the effects of compressive cryotherapy on postoperative pain levels and knee swelling in patients undergoing knee arthroscopy.

The findings of the present study demonstrated a significant and rapid reduction in pain intensity among patients receiving compressive cryotherapy compared with those in the control group following knee arthroscopy. This result is consistent with previous studies supporting the effectiveness of compressive cryotherapy in the management of postoperative pain and swelling. Notably, a marked analgesic effect was observed within 15 min of application, indicating a rapid onset of action. This finding aligns with another study involving patients after hip arthroscopy, which reported significantly lower pain scores in patients treated with compressive cryotherapy following the procedure [26]. The rapid pain relief may be explained by the synergistic effects of cold therapy and compression, which enhance tissue cooling, reduce local inflammation, and augment the analgesic effects beyond those achieved with cryotherapy alone. Moreover, Quesnot et al. (2024) randomized controlled trial after total knee arthroplasty reported that adding compressive cryotherapy to standard rehabilitation accelerated improvement in knee circumference, pain during activity, and some functional outcomes compared with standard cryotherapy alone [14].

The sustained reduction in pain observed in the compressive cryotherapy group during the first and second postoperative days is consistent with the meta-analysis in 2016, which reported lower pain levels in patients receiving compressive cryotherapy compared with cryotherapy alone during the early postoperative period following knee surgery [22]. This prolonged analgesic effect suggests that compressive cryotherapy may provide more consistent and effective pain control throughout the critical early recovery phase. The significant differences in pain intensity between the compressive cryotherapy and control groups on both postoperative days further highlight the potential of compressive cryotherapy as an effective non-pharmacological pain management strategy. This finding is particularly relevant in the context of ongoing efforts to reduce opioid consumption in postoperative care. By effectively alleviating pain without exclusive reliance on pharmacological interventions, compressive cryotherapy may contribute to enhanced recovery outcomes and potentially shorter hospital stays, as suggested by previous research in patients undergoing total knee arthroplasty [27].

The progressive shift toward milder pain categories observed in the compressive cryotherapy group over time, with no patients reporting severe pain by the first postoperative day, is particularly encouraging. This pattern suggests that compressive cryotherapy may facilitate earlier mobilization and rehabilitation, which are critical for preventing postoperative complications such as joint stiffness or capsulolabral adhesions, as reported in studies of hip arthroscopy patients [19]. Although pain levels also improved over time in the control group, the magnitude and rate of improvement were less pronounced than those observed in the compressive cryotherapy group. These differences in pain reduction trajectories further support the potential value of incorporating compressive cryotherapy into postoperative care protocols for patients undergoing knee arthroscopy.

The statistically significant differences observed between the groups across all time points provide strong evidence supporting the superiority of compressive cryotherapy over standard cryotherapy in managing postoperative pain. These findings are consistent with the growing body of literature advocating the use of compressive cryotherapy to enhance recovery following orthopedic surgery [27,28]. A more recent 2025 narrative review on cryotherapy mechanisms emphasized that cold-induced reductions in nerve conduction velocity, local blood flow, and inflammatory mediator release, especially when enhanced by external compression, are key mechanisms underlying the rapid analgesic and antiedematous effects seen clinically [29].

The current study results reveal a significant reduction in knee circumference measurements for patients receiving compressive cryotherapy compared to the control group following knee arthroscopy. This finding aligns with previous research on the efficacy of compressive cryotherapy in managing postoperative swelling and edema [30,31,32,33]. The immediate postoperative measurements show no significant difference between the two groups, indicating a comparable baseline of postoperative swelling. This similarity at the outset strengthens the validity of the subsequent comparisons, as it suggests that any differences observed later are likely due to the intervention rather than pre-existing variations between the groups.

The marked reduction in knee circumference observed in the compressive cryotherapy group by the first postoperative day is particularly noteworthy. This rapid decrease in swelling supports the findings of Murgier and Cassard (2014), who reported significantly faster improvement in knee circumference for patients receiving compressive cryotherapy after total knee arthroplasty [28]. The reduction in swelling observed with compressive cryotherapy can be attributed to enhanced lymphatic drainage and reduced vascular permeability. Compression aids in mechanical fluid displacement from the interstitial space, while cold-induced vasoconstriction limits capillary leakage and inflammatory exudate. This synergistic effect promotes faster resolution of edema compared to cryotherapy alone, as supported by recent physiological studies [17]. The continued decrease in knee circumference for the compressive cryotherapy group on the second postoperative day, contrasted with the minimal change in the control group, underscores the sustained benefits of compressive cryotherapy over time [28]. This prolonged effect is consistent with the meta-analysis study, which found that patients undergoing compressive cryotherapy tended to have less swelling than those receiving cryotherapy alone in the early postoperative period following knee surgery [22].

The significant differences observed between the groups at all time points, as indicated by the Mann–Whitney U test results, provide strong evidence for the superiority of compressive cryotherapy over standard cryotherapy in managing postoperative swelling. These findings align with the growing body of literature supporting the use of compressive cryotherapy in orthopedic surgery recovery, including studies on hip arthroscopy patients [27] and knee replacement surgery patients [10]. The substantial reduction in swelling observed in the compressive cryotherapy group could have important clinical implications. Decreased swelling may contribute to reduced pain, improved range of motion, and potentially earlier mobilization. As Yang et al. (2023) highlighted, effective management of postoperative swelling is crucial for optimizing patient outcomes and facilitating early functional recovery [34]. Compressive cryotherapy’s ability to significantly reduce swelling without relying solely on pharmacological interventions could improve rehabilitation outcomes and potentially shorten hospital stays.

The therapeutic effect of cryotherapy in post-arthroscopic care is primarily attributed to its ability to lower intra-articular temperature, thereby reducing the local inflammatory response. Research has shown that the application of ice with compression after knee arthroscopy significantly decreases intra-articular temperature, whereas untreated knees tend to exhibit an early postoperative increase in joint temperature. This cooling effect on synovial and periarticular tissues supports the physiological basis for the clinical benefits of cryotherapy, particularly in reducing pain and swelling, and is consistent with its established anti-inflammatory and analgesic properties [8,35]. A recent systematic review reported that cryocompression devices are more effective than traditional icing alone in reducing postoperative pain and narcotic use following arthroscopic knee surgery. However, they were found to be no more effective than compression alone [29].

Furthermore, the statistically significant changes in knee circumference over time within both groups, as indicated by the Friedman test results, suggest that both interventions have some effect on reducing swelling. However, the magnitude of these changes differed substantially between the groups, with the compressive cryotherapy group showing more pronounced and consistent reductions. This difference in swelling reduction trajectories further emphasizes the potential benefits of incorporating compressive cryotherapy into postoperative care protocols for knee arthroscopy patients.

### Study Limitations

A key limitation of this study is its quasi-experimental design, which lacks randomization. The sequential, non-random allocation of participants introduces the potential for selection bias and temporal confounding, wherein unmeasured factors related to the timing of enrollment, such as seasonal variations in hospital protocols or changes in surgical teams, could influence outcomes. Although we statistically confirmed baseline equivalence on measured variables and conducted sensitivity analyses, the inability to randomize remains a constraint on causal inference. Future studies should consider stratified recruitment or randomization across seasons and surgical teams to mitigate such biases. Furthermore, the use of non-parametric tests, while appropriate for our data distribution, may have reduced statistical power compared to parametric alternatives. Future studies with larger sample sizes could employ mixed-methods analytical approaches to further validate these findings. The relatively short follow-up period limits the ability to assess the long-term effects of compressive cryotherapy on recovery, such as functional recovery and range of motion. Additionally, the study was conducted in a single setting, which may affect the generalizability of the findings to broader populations. Lastly, factors such as variations in individual pain tolerance and adherence to postoperative care could influence outcomes, suggesting a need for further research to explore these variables in diverse clinical contexts.

## 7. Conclusions

This study demonstrates that compressive cryotherapy significantly reduces postoperative pain intensity and knee circumference in patients undergoing knee arthroscopy compared with standard care. Patients who received compressive cryotherapy showed a marked reduction in pain and swelling, particularly during the first two postoperative days. These findings support the effectiveness of compressive cryotherapy as a beneficial intervention for enhancing postoperative recovery and improving patient comfort, indicating its potential for routine use in clinical practice following knee arthroscopy.

### Nursing Implications

Integrating compressive cryotherapy following knee arthroscopy into routine care may help reduce postoperative pain and swelling. In addition, providing patients with educational materials, such as posters and illustrated pamphlets, can support the correct administration of postoperative compressive cryotherapy.

## Figures and Tables

**Table 1:** The demographic and Clinical data of the participants.

Demographic Data	Intervention Group (*n* = 30)		Control Group (*n* = 30)		χ^2^	^MC^ *p*
	No	%	No	%		
**Age**						
20–29	2	6.7%	3	10.0%	3.848	0.495
30–39	8	26.7%	8	26.7%		
40–49	11	36.7%	10	33.3%		
50–59	9	30.0%	9	30.0%		
**Gender**						
Male	14	46.7%	10	33.3%	2.443	0.192
Female	16	53.3%	20	66.7%		
**Level of Educational**						
Cannot read or write	17	56.7%	12	40.0%	3.973	0.29
Primary education	5	16.7%	4	13.3%		
Secondary education	3	10.0%	9	30.0%		
Bachelor’s degree	5	16.7%	5	16.7%		
**Marital status**						
Single	4	13.3%	6	20.0%	2.9	0.435
Married	17	56.7%	17	56.7%		
Widowed	6	20.0%	2	6.7%		
Divorced	3	10.0%	5	16.7%		
**Employment**						
Unemployed	14	46.7%	10	33.3%	1.314	0.518
Physical job	10	33.3%	11	36.7%		
Sedentary job	6	20.0%	9	30.0%		
**Region of Residence**						
Rural	9	30.0%	9	30.0%	0.000	1.000
Urban	21	70.0%	21	70.0%		
**Monthly Income**						
Insufficient	18	60.0%	17	56.7%		
Sufficient	12	40.0%	13	43.3%	3.354	0.067
**Comorbidities**						
Yes	11	36.7%	11	36.7%	0.000	1.000
No	19	63.3%	19	63.3%		
**Diagnosis and present conditions**						
KA for diagnostic purposes	8	26.7%	9	30.0%	0.000	1.000
KA for therapeutic purposes	22	73.3%	21	70.0%		
**Previous knee surgeries**						
Yes	9	30.0%	8	26.7%	0.082	0.774
No	21	70.0%	22	73.3%		
**Previous family history of orthopedic disorders**						
Yes	12	40.0%	12	40.0%	0.000	1.000
No	18	60.0%	18	60.0%		
**BMI**						
**Normal weight:** BMI (**18.5—24.9 kg/m^2^**)	6	20.0%	4	13.3%	0.622	0.733
**Overweight:** BMI (**25.0—29.9 kg/m^2^**)	8	26.7%	9	30.0%		
**Obese:** BMI (**≥30.0 kg/m^2^**)	16	53.3%	17	56.7%		

χ^2^: Chi-square test; ^MC^: Monte Carlo; *: Statistically significant at *p* ≤ 0.05.

**Table 2:** Pain Intensity Categories Across Postoperative Time Points.

NRS-11	Intervention Group (*n* = 30)		Control Group (*n* = 30)		χ^2^	^MC^ *p*
	No	%	No	%		
**T0: Immediately after the surgery (Baseline)**						
Mild	0	0.0%	0	0.0%		
Moderate	0	0.0%	0	0.0%	-	-
Severe	30	100.0%	30	100.0%		
**T0 + 15 min: After the surgery**						
Mild	2	6.7%	1	3.3%		
Moderate	20	66.7%	5	16.7%	30.127 *	<0.001 *
Severe	8	26.6%	24	80.0%		
**T1: 1st day after the surgery**						
Mild	9	30.0%	1	3.3%		
Moderate	21	70.0%	8	26.7%	33.228 *	<0.001 *
Severe	0	0.0%	21	70.0%		
**T2: 2nd day after the surgery**						
Mild	17	56.7%	8	26.7%		
Moderate	13	43.3%	8	26.7%	18.430 *	<0.001 *
Severe	0	0.0%	14	46.7%		
**Fr. (p)**	56.408 * (<0.001) *		22.262 * (<0.001) *			
**P_1_**	<0.001 *		0.061			
**P_2_**	<0.001 *		0.001 *			
**P_3_**	0.302		0.138			

NRS-11: Numerical Rating Scale-11; χ^2^: Chi-square test; MC: Monte Carlo simulation. Within-group changes in pain intensity across time points were analyzed using the Friedman test. Significant findings were further examined with post hoc pairwise comparisons using the Wilcoxon signed-rank test, adjusted with the Bonferroni–Holm method. P_1_: T0 vs. T0 + 15 min; P_2_: T0 vs. T2; P_3_: T1 vs. T2. * *p* < 0.05.

**Table 3:** Knee Circumference Across Postoperative Time Points.

Knee Circumference Measurements (cm)	Intervention Group (*n* = 30)	Control Group (*n* = 30)	U (Z)	*p*
	Median [IQR]	Median [IQR]		
**T0: Immediately after the surgery (Baseline)**	51.05 [49.1–53.2]	52.70 [50.8–54.5]	382.5 (−0.86)	0.390
**T0 + 15 min: After first session**	42.20 [40.5–44.1]	50.10 [48.3–52.0]	105.0 (−5.94) *	<0.001 *
**T2: 2nd day after surgery**	40.90 [39.8–42.1]	48.55 [46.8–50.9]	62.5 (−6.72) *	<0.001 *
**Within-group test χ^2^(2)**	58.32, *p* < 0.001 *	28.15, *p* < 0.001 *		
**P_1_**	<0.001 *	0.003 *		
**P_2_**	<0.001 *	0.001 *		
**P_3_**	<0.001 *	0.002 *		

U: Mann–Whitney U test statistic; Z: standardized test statistic derived from the Mann–Whitney U; **χ^2^(2)** = Friedman test statistic with 2 df. Post hoc pairwise comparisons for within-group changes were conducted using the Wilcoxon signed-rank test, with *p*-values adjusted via the Bonferroni–Holm correction. P_1_: T0 vs. T0 + 15 min; P_2_: T0 vs. T2; P_3_: T1 vs. T2. * *p* < 0.05.

## Data Availability

The data presented in this study are available from the corresponding author upon reasonable request, subject to approval from the affiliated institution.
